# Editorial for the Special Issue “Imaging Diagnosis in the Abdomen”—A Step Forward in Diagnostic Precision

**DOI:** 10.3390/diagnostics15050557

**Published:** 2025-02-25

**Authors:** Piero Boraschi, Francescamaria Donati

**Affiliations:** 2nd Unit of Radiology, Department of Radiological Nuclear and Laboratory Medicine, Pisa University Hospital, via Paradisa 2, 56124 Pisa, Italy; f.donati@med.unipi.it

Abdominal imaging has undergone a significant transformation in recent years, driven by the rapid evolution of diagnostic technologies and their integration into clinical practice. With an expanding range of advanced imaging techniques—such as MRI with hepatobiliary contrast agents [1], diffusion and perfusion imaging [2], contrast-enhanced ultrasound [3], and spectral imaging via dual-energy, multi-energy, and photon-counting CT [4,5]—there has been a revolution in disease detection, staging, and treatment planning. These innovations have greatly enhanced diagnostic accuracy and patient care, particularly in the management of complex abdominal diseases.

Despite these advancements, significant gaps remain in our understanding of certain disease processes and the optimal utilization of emerging imaging modalities. One of the key challenges moving forward is the need for further validation and standardization of newer techniques, such as perfusion imaging and spectral CT, across diverse clinical settings. Additionally, while the potential of advanced imaging in assessing tumor vascularity and predicting prognosis is promising, more research is required to fully leverage these capabilities in personalized treatment planning [6].

The contributions to this Special Issue on *Imaging Diagnosis in the Abdomen* have addressed some of these gaps by presenting the latest advancements in imaging technology and its application in the diagnosis of complex abdominal conditions. For example, studies like Wang et al.’s exploration of multi-slice CT for predicting the pathological risk of gastric stromal tumors [7] and Zaborienė et al.’s use of dynamic contrast-enhanced MRI for assessing pancreatic cancer [8] highlight the ongoing progress in tumor characterization and risk stratification. However, while these studies underscore the promise of advanced imaging techniques, they also point to areas where additional research is needed, particularly in improving the accuracy of tumor grading and enhancing non-invasive diagnostic strategies.

Looking forward, the future of abdominal imaging will likely be shaped by further innovations in artificial intelligence (AI) and machine learning (ML) [9,10]. These technologies have the potential to automate image analysis, improve diagnostic accuracy, and facilitate real-time decision-making. In particular, AI and ML algorithms could play a critical role in integrating multimodal imaging data, enabling clinicians to generate more holistic assessments of disease. Moreover, the increasing role of imaging in monitoring therapeutic responses, particularly in immunotherapy [11,12], will require new frameworks to assess treatment efficacy and tailoring interventions.

While this Special Issue highlights the progress made in abdominal imaging, it also emphasizes the need for continued research to optimize imaging techniques for early disease detection, enhance diagnostic workflows, and improve patient outcomes. Future studies should focus on the development of more refined imaging biomarkers, the greater standardization of imaging protocols, and the potential applications of artificial intelligence in clinical radiology. These efforts will undoubtedly be pivotal in advancing the field and addressing the unmet needs in abdominal imaging.

The research presented in this Special Issue will continue to serve as a valuable resource, laying the foundation for future investigations and shaping the direction of abdominal imaging in the years to come.
